# Melatonin Exerts Cardioprotective Effects by Inhibiting NLRP3 Inflammasome-Induced Pyroptosis in Mice following Myocardial Infarction

**DOI:** 10.1155/2021/5387799

**Published:** 2021-09-01

**Authors:** Lianghe Wen, Minnan Wang, Peiyao Luo, Xianglin Meng, Mingyan Zhao

**Affiliations:** ^1^Department of Critical Care Medicine, The Second Affiliated Hospital of Harbin Medical University, No. 246 Xuefu Road, Harbin, 150086 Heilongjiang Province, China; ^2^Department of Endocrinology and Metabolism, The Second Affiliated Hospital of Harbin Medical University, No. 246 Xuefu Road, Harbin, 150086 Heilongjiang Province, China; ^3^Department of Critical Care Medicine, The First Affiliated Hospital of Harbin Medical University, No. 23 Youzheng Street, Harbin, 150001 Heilongjiang Province, China

## Abstract

Myocardial infarction- (MI-) induced myocardial damage is mainly attributed to the loss of cardiomyocytes. Pyroptosis is a newly recognized form of programmed cell necrosis that is associated with the progression of MI. Melatonin has been shown to exert cardioprotective effects against cardiac damage in multiple cardiovascular diseases. However, the effect of melatonin on pyroptosis-induced cardiac injury in MI has not been elucidated. Herein, we found that melatonin administration ameliorated cardiac dysfunction and reduced cardiomyocyte death both in mice following coronary artery ligation and in H9C2 cells exposed to hypoxia. The results also showed that pyroptosis was induced both *in vivo* and *in vitro*, as evidenced by increased NLRP3, cleaved caspase-1, GSDMD-N, and mature IL-1*β* and IL-18 levels, and these changes were decreased by melatonin treatment. Furthermore, we observed that TLR4 and NF-*κ*B levels were increased by MI or hypoxia, and these increases were reversed by melatonin. The antipyroptotic action of melatonin was abrogated by treatment with an agonist of the TLR4/NF-*κ*B signaling pathway. Our results indicate that melatonin can exert cardioprotective effects by inhibiting NLRP3 inflammasome-induced pyroptosis through modulation of the TLR4/NF-*κ*B signaling pathway and provide strong evidence for the utility of melatonin in the treatment of MI.

## 1. Introduction

Myocardial infarction (MI) is a leading cause of morbidity and mortality worldwide. It is widely acknowledged that MI can deteriorate left ventricle cardiomyocytes and aggravate cardiac remodeling, eventually resulting in heart failure [1–3]. Cardiomyocyte death occurs at the onset of MI and ultimately contributes to myocardial damage [4, 5]. Although reperfusion therapy is an effective strategy to reduce infarct size, it also potentiates myocardial damage, known as ischemia-reperfusion injury [6, 7]. Thus, the identification of effective and innovative therapies for reducing cardiomyocyte loss may play a critical role in the treatment of MI.

Pyroptosis is a type of programmed cell necrosis mediated by the gasdermin family. Inflammasomes, which are multiprotein complexes, are activated under distinct pathological stimuli and subsequently trigger the activation of caspase-1. On the one hand, active caspase-1 contributes to pyroptosis by cleaving gasdermin D (GSDMD) to produce an N-terminal fragment of GSDMD (GSDMD-N), which is widely considered a pyroptosis executor. On the other hand, active caspase-1 also leads to an inflammatory response by inducing interleukin (IL)-1*β* or IL-18 maturation [8–11]. Recent studies have demonstrated that pyroptosis is involved in cardiomyocyte loss induced by MI [12, 13]. The nucleotide-binding oligomerization domain-like receptor family pyrin domain-containing 3 (NLRP3) inflammasome, one of the best-known inflammasomes, was found to be activated upon MI and to induce cardiomyocyte pyroptosis by triggering caspase-1 and cleaved GSDMD [14, 15]. However, inhibition of the NLRP3 inflammasome by MCC950 significantly reduced infarct size and improved cardiac remodeling [16]. Inhibition of pyroptosis could ameliorate the damage to cardiomyocytes caused by MI [17]. These studies indicate that targeting NLRP3 inflammasome-mediated pyroptosis may be an effective strategy for reducing MI-induced myocardial injury.

The neuroendocrine hormone melatonin has multiple effects, including regulating circadian rhythm and immunity; influencing sleep; delaying aging via effects against oxidation, apoptosis, fibrosis, and autophagy; and protecting against mitochondria and endoplasmic reticulum stress [18]. It has been widely demonstrated that melatonin has a cardioprotective effect in MI [19]. For example, melatonin can ameliorate cardiac remodeling by activating the Notch1/Mfn2 pathway in the hearts of mice following MI [20]. Melatonin exerts anti-inflammatory activity by inhibiting the release of inflammatory cytokines, including IL-1*β*, IL-18, TNF-*α*, C-reactive protein, Nrf2, and IL-1*α*, during the development of MI [21, 22]. However, whether melatonin can regulate cardiomyocyte pyroptosis in MI remains unclear.

The aim of this study was to evaluate the possible effect of melatonin on cardiomyocyte pyroptosis in MI and to further identify the underlying molecular mechanisms. Our results demonstrated that melatonin alleviated cardiomyocyte loss by inhibiting NLRP3 inflammasome-mediated pyroptosis by modulating the Toll-like receptor 4 (TLR4)/NF-*κ*B signaling pathway and further clarified that melatonin may be a promising agent for MI treatment.

## 2. Methods and Materials

### 2.1. Animals

Male C57BL/6 mice (weighing 20 ± 2 g) were obtained from The Second Affiliated Hospital of Harbin Medical University. This study was approved by the Animal Protection and Use Committee of Harbin Medical University and was performed in line with the recommendations in the Guide for the Care and Use of Laboratory Animals issued by the National Institutes of Health.

### 2.2. MI Model and Drug Treatment

The mice were randomly divided into three groups: the sham group (*n* = 12), the MI group (MI, *n* = 12), and the melatonin treatment group (MI+MLT, *n* = 12). The MI model was generated with a method similar to that in previous studies [23, 24]. Briefly, the mice were anaesthetized by avertin (0.2 g/kg) injection. The left anterior descending coronary artery was exposed and ligated with a 7-0 nylon suture for 24 h. The mice in the sham group underwent the same surgical procedures without coronary artery ligation. The mice in the MI+MLT group were administered 10 mg/kg melatonin (M5250, Sigma-Aldrich) per day by intragastric gavage for 14 days prior to MI surgery [25]. The mice in the sham and MI groups were given an equivalent volume of ddH_2_O.

### 2.3. Echocardiography

Cardiac function was detected by using an ultrasound system (VisualSonics, Toronto, ON, Canada) after MI for twenty-four hours. The mice were anaesthetized by avertin injection and fixed onto a flat plate. After exposing the chest, left ventricular systolic diameter (LVDs), left ventricular diastolic diameter (LVDd), left ventricular ejection fraction (EF), and left ventricular fractional shortening (FS) were detected and analyzed.

### 2.4. Western Blot Assay

Tissues or cells were fully dissolved in RIPA buffer (P0013B, Beyotime, China) containing protease inhibitor (4693159001, Roche, Germany) and phosphatase inhibitor (4906837001, Roche, Germany). Sample were separated by SDS-PAGE gel and then transferred onto a nitrocellulose membrane. After the membranes were blocked with 5% nonfat milk, they were incubated at 4°C overnight with the following antibodies: NLRP3 (1 : 500, bs-10021R, Bioss), cleaved caspase-1 (1 : 200, ab207802, Abcam), GSDMD-N (1 : 200, ab215203, Abcam, USA), TLR4 (1 : 200, sc-293072, Santa Cruz Biotechnology), NF-*κ*B (p-65, 1 : 200, sc-8008, Santa Cruz Biotechnology), IL-1*β* (1 : 200, ab254360, Abcam), IL-18 (1 : 100, ab207324, Abcam), and *β*-actin (1 : 2000, TA-09, ZSGB). Then, the secondary antibody was added, and the membrane was incubated at room temperature. After a washing step, the gray values were evaluated by Quantity One software and subjected to statistical analysis.

### 2.5. Real-Time PCR Assay

Cells and tissues were fully lysed with TRIzol (15596026, Invitrogen, USA) according to manufacturer's instructions. The RNA concentration was determined by a NanoDrop instrument. A reverse transcription kit (FSQ-101, Toyobo) was used to synthesize cDNA. Real-time PCR was performed using an ABI 7500 Fast PCR instrument with the SYBR Green Real-time PCR Master Kit (QPK-201, Toyobo). The primer sequences were as follows: NLRP3 (forward: 5′-CAACCTCACGTCACACTGCT-3′; reverse: 5′-TTTCAGACAACCCCAGGTTC-3′), caspase-1 (forward: 5′-ACACGTCTTGCCCTCATTATCT-3′; reverse: 5′-ATAACCTTGGGCTTGTCTTTCA-3′), and GAPDH (forward: 5′-AAGAAGGTGGTGAAGCAGGC-3′; reverse: 5′-TCCACCACCCAGTTGCTGTA-3′). GAPDH was used to normalize target expression levels. RNA expression levels were computed by the 2^−△△CT^ method.

### 2.6. Cell Culture, Drug Treatment, and Hypoxic Conditions

H9C2 cells were purchased from American Type Culture Collection (Manassas, USA) and cultured in Dulbecco's modified Eagle's medium (DMEM, SH30022.01, HyClone) supplemented with 10% fetal calf serum and 1% penicillin-streptomycin in an incubator (5% CO_2_, 95% O_2_, 37°C). When the cell density reached 70-80%, the cells were moved into a hypoxic chamber (95% N_2_, 5% O_2_, 37°C) and incubated for 120 min with or without melatonin (10 *μ*M, M5250, Sigma-Aldrich) [26].

### 2.7. Immunofluorescence Staining

Treated or untreated H9C2 cells were fixed with 4% paraformaldehyde. After a blocking step, anti-NLRP3 (1 : 100, bs-10021R, Bioss) or anti-TLR4 (1 : 100, sc-293072, Santa Cruz Biotechnology) was added, and the cells were incubated at 4°C overnight. Then, secondary antibody conjugated to Alexa Fluor 488 (A11034, Invitrogen) was added for 1 h at room temperature. Finally, the nucleus was stained with 4′,6-diamino-2-phenylindole (DAPI, C0065, Solarbio). All images were acquired under a fluorescence microscope (Nikon 80i, Japan).

### 2.8. Immunohistochemical Staining

The heart tissues were removed, fixed, and embedded in paraffin. After a blocking step, a primary anti-TLR4 antibody (1 : 100, sc-293072, Santa Cruz Biotechnology) was added, and the tissues were incubated overnight at 4°C. The next day, the secondary antibody was added for 20 min at room temperature. The images were captured by a microscope (Zeiss, Germany).

### 2.9. Calcein AM/EthD-III Staining

Calcein AM/EthD-III staining was performed according to manufacturer's instructions (30002, Biotium, USA). Briefly, untreated and treated H9C2 cells were washed three times with PBS, and then, 200 *μ*L of calcein-AM/EthD-III staining solution was added. After incubation for 40 min, images of the cells were captured by using a fluorescence microscope (Nikon 80i, Japan).

### 2.10. Lactate Dehydrogenase (LDH) Release Assay

The supernatants from different groups were collected, and LDH was detected using an LDH assay kit (A020-2-2, Nanjingjiancheng, China) according to manufacturer's instructions. A microplate reader was used to analyze the absorbance value of each sample at 450 nm.

### 2.11. Hematoxylin and Eosin (HE) Staining

Heart tissues were fixed and embedded in paraffin. After cutting the paraffin tissue blocks into 5 *μ*m thick sections, HE staining was performed by using an HE kit (G1120, Solarbio) according to manufacturer's instructions. Images were collected under a microscope (Zeiss, Germany).

### 2.12. TUNEL Staining

Heart tissues from different groups were embedded in paraffin and then cut into 5 *μ*m sections. The In Situ Cell Death Detection Kit (11684817910, Roche, Germany) was used to detect cell death following manufacturer's instructions with some modifications. After deparaffinization, the TUNEL reaction mixture was added to the paraffin sections, which were then incubated for 1 h at 37°C. The nuclei were stained with DAPI (C0065, Solarbio) for 15 min at room temperature. Fluorescence microscopy was used to capture the images (Nikon 80i, Japan).

### 2.13. Statistical Analysis

The data are presented as the mean ± SEM. One-way ANOVA was used to compare the differences between groups with SPSS 22.0 software. *P* < 0.05 was considered to indicate statistical significance.

## 3. Results

### 3.1. Melatonin Alleviates Cardiac Injury in MI Mice

**Figure 1:**
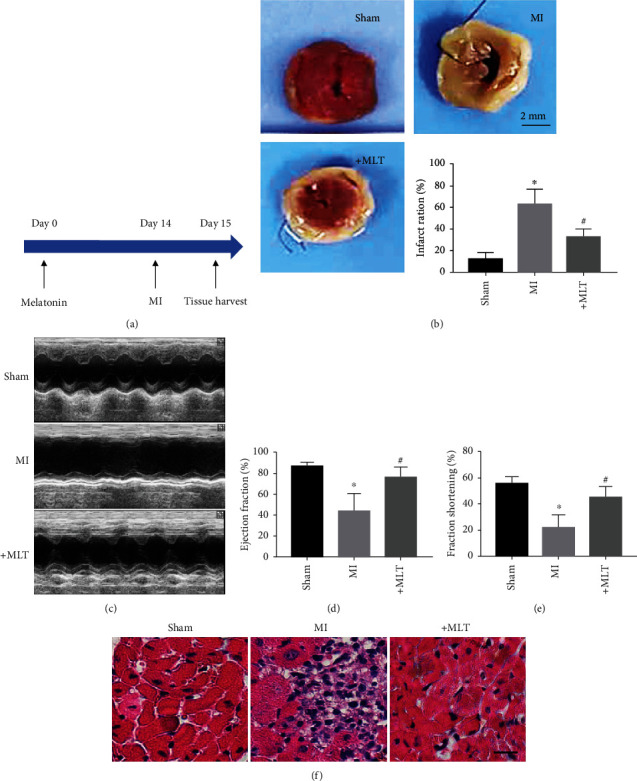
Cardiac injury was reversed by melatonin treatment in MI mice. (a) Diagram of the experimental protocol. (b) Representative LV sections stained with TTC and statistical analysis of infarct area data in mice. The infarct region is labeled in white. ^∗^*P* < 0.05 vs. sham, ^#^*P* < 0.05 vs. MI. Number of trials = 5‐6. (c) Representative echocardiography images from different groups. (d, e) Statistical analysis of echocardiographic EF% and FS% data in MI mice treated with or without melatonin. ^∗^*P* < 0.05 vs. sham, ^#^*P* < 0.05 vs. MI. Number of trials = 6. (f) Representative images of H&E staining of the mouse heart. Scale bar, 20 *μ*m. MI: myocardial infarction; MLT: melatonin.

To determine whether melatonin has cardioprotective effects after MI, we established a mouse model of MI and pretreated the mice with melatonin for 14 days prior to coronary artery ligation (Figure 1(a)). TTC staining was used to measure the infarct area 24 h after MI. The infarct area was markedly increased in the MI group compared with the sham group, while melatonin administration significantly decreased the infarct area induced by MI (Figure 1(b)). As shown in Figures 1(c)–1(e), the echocardiographic data showed that the EF and FS values were significantly lower in MI mice than in sham mice, suggesting that cardiac damage was induced by MI in mice. Notably, the EF and FS values were both effectively restored by melatonin administration in MI mice. Additionally, the melatonin-treatment group had reduced infiltration of inflammatory cells compared with the MI group (Figure 1(f)).

### 3.2. Melatonin Attenuates NLRP3 Inflammasome-Mediated Cardiac Pyroptosis in MI Mice

**Figure 2:**
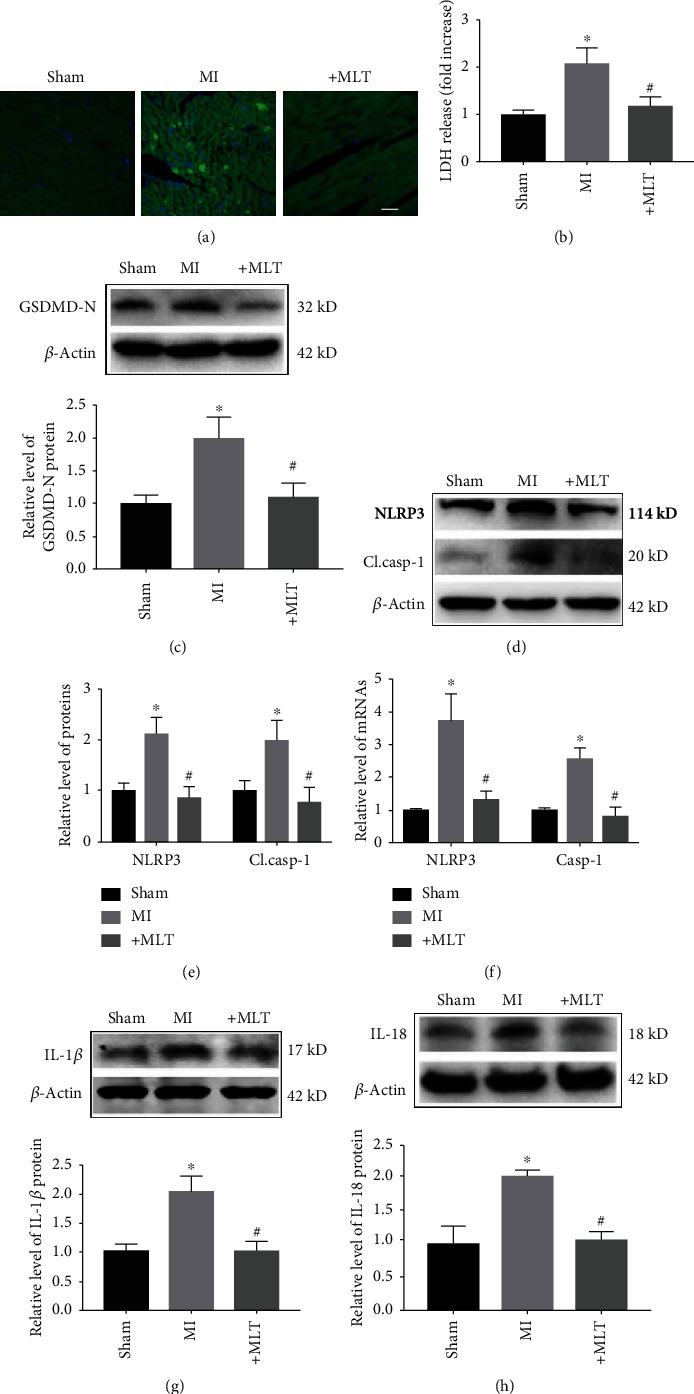
Effect of melatonin on NLRP3 inflammasome-mediated cardiac pyroptosis in MI mice. (a) Representative images from TUNEL staining in different groups. Scale bar, 20 *μ*m. (b) Melatonin treatment decreased the release of LDH in MI mice. ^∗^*P* < 0.05 vs. sham, ^#^*P* < 0.05 vs. MI. Number of trials = 6. (c) The level of GSDMD-N was decreased by melatonin treatment. ^∗^*P* < 0.05 vs. sham, ^#^*P* < 0.05 vs. MI. Number of trials = 5. (d, e) Melatonin treatment reduced the levels of NLRP3 and cl.casp-1 in the hearts of MI mice. ^∗^*P* < 0.05 vs. sham, ^#^*P* < 0.05 vs. MI. Number of trials = 5‐6. (f) The mRNA levels of NLRP3 and caspase-1 in the different groups were detected by real-time PCR. ^∗^*P* < 0.05 vs. sham, ^#^*P* < 0.05 vs. MI. Number of trials = 6. (g, h) The levels of IL-1*β* and IL-18 in heart tissue from different groups. ^∗^*P* < 0.05 vs. sham, ^#^*P* < 0.05 vs MI. Number of trials = 4‐5. MI: myocardial infarction; cl.casp-1: cleaved caspase-1; MLT: melatonin.

Previous studies have demonstrated that pyroptosis contributes to cardiomyocyte loss in the development of MI [27, 28]. Therefore, we next determined whether the cardioprotective effect of melatonin in the hearts of MI mice was associated with pyroptosis. TUNEL staining showed that melatonin treatment reversed the MI-induced cardiomyocyte loss (Figure 2(a)). Moreover, LDH levels were significantly increased in the serum of MI mice, suggesting that the plasma membrane was ruptured. Importantly, melatonin administration decreased LDH levels (Figure 2(b)). As shown in Figure 2(c), the level of GSDMD-N, a pyroptosis executor, was significantly increased in the hearts of MI mice compared with sham mice; however, this increase was reversed by melatonin treatment. These data suggest that melatonin can inhibit pyroptotic cardiomyocyte death induced by MI.

The NLRP3 inflammasome has been shown to be involved in cell death by inducing pyroptosis, and inhibition of the NLRP3 inflammasome exerts protective effects in various diseases [29–31]. To ascertain whether the antipyroptotic effect of melatonin in MI is associated with the NLRP3 inflammasome, we detected changes in this large multiprotein complex. In this study, we found that the protein and mRNA levels of NLRP3 and cleaved caspase-1 were decreased in the MI+MLT group compared with the MI group (Figures 2(d)–2(f)). Caspase-1 activation is known to lead to cardiac damage by inducing the maturation of IL-1*β* or IL-18. Thus, we also detected the effect of melatonin on IL-1*β* and IL-18 in heart tissue. As shown in Figures 2(g) and 2(h), the protein levels of IL-1*β* and IL-18 were increased by MI but were reduced by melatonin administration.

### 3.3. Melatonin Inhibits NLRP3 Inflammasome-Mediated Cardiac Pyroptosis *In Vitro*

**Figure 3:**
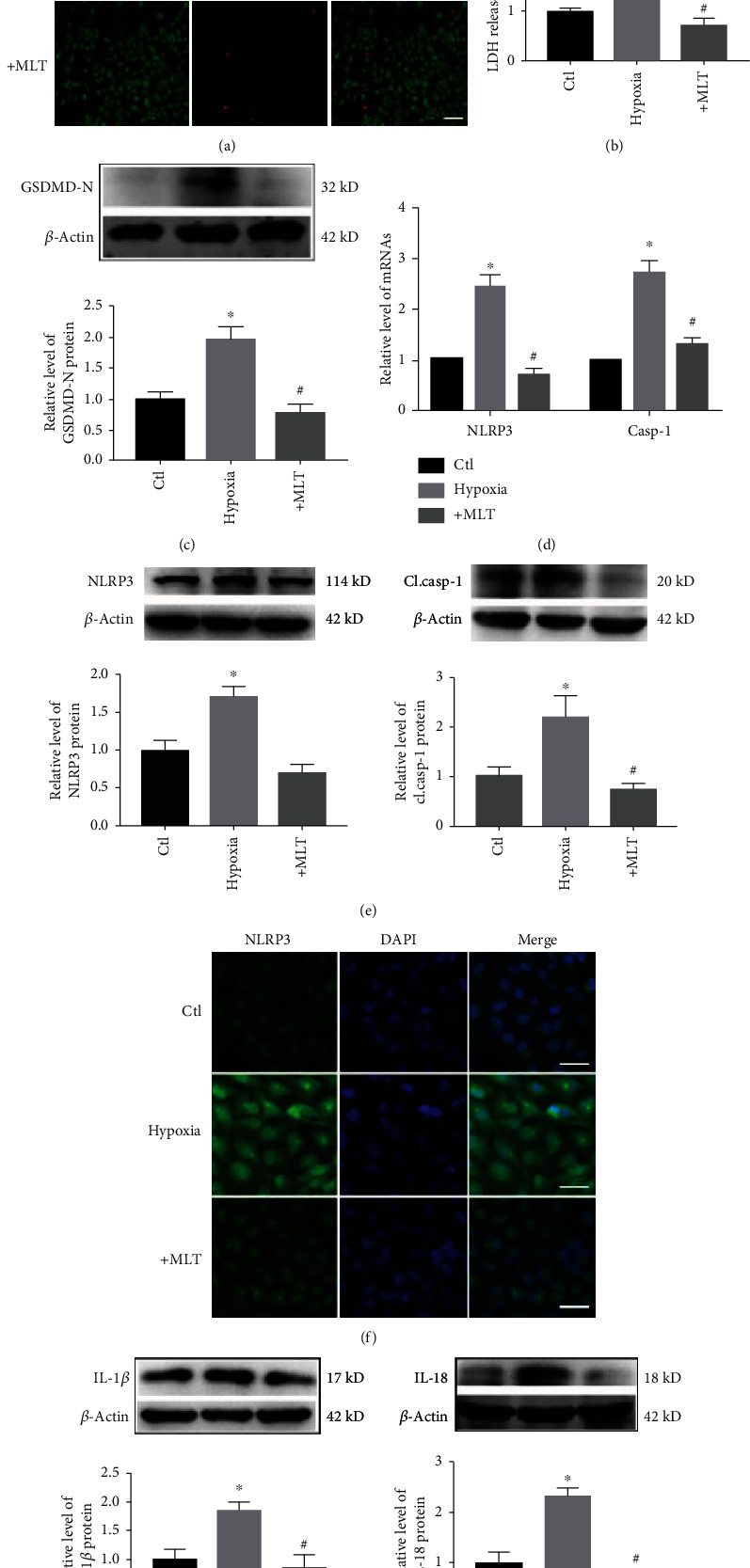
Effect of melatonin on NLRP3 inflammasome-mediated cardiac pyroptosis in H9C2 cells exposed to hypoxia. (a) Melatonin reduced hypoxia-induced cardiomyocyte loss. Live cells were stained with calcein-AM (green), and dead cells were stained with EthD-III (red). Scale bar, 40 *μ*m. (b) Melatonin treatment decreased the release of LDH in H9C2 cells. ^∗^*P* < 0.05 vs. ctl, ^#^*P* < 0.05 vs. hypoxia. Number of trials = 3. (c) GSDMD-N levels were decreased by melatonin treatment. ^∗^*P* < 0.05 vs. ctl, ^#^*P* < 0.05. Number of trials = 3. (d) The mRNA levels of NLRP3 and caspase-1 in the different groups were detected by real-time PCR. ^∗^*P* < 0.05 vs. ctl, ^#^*P* < 0.05. Number of trials = 3. (e) Melatonin treatment reduced the levels of NLRP3 and cl.casp-1 in hypoxia-treated H9C2 cells. ^∗^*P* < 0.05 vs. ctl, ^#^*P* < 0.05 vs. hypoxia. Number of trials = 3. (f) NLRP3 levels were detected by immunofluorescence. Scale bar, 50 *μ*m. (g) The levels of IL-1*β* and IL-18 were decreased by melatonin in H9C2 cells. ^∗^*P* < 0.05 vs. ctl, ^#^*P* < 0.05 vs. hypoxia. Number of trials = 3. cl.casp-1: cleaved caspase-1; MLT: melatonin.

Next, we aimed to determine whether melatonin can attenuate NLRP3 inflammasome-mediated cardiac pyroptosis *in vitro*. Hypoxia plays a critical role in the onset of MI; thus, we established an *in vitro* model of hypoxia in H9C2 cardiac cells to mimic MI conditions. We found that melatonin significantly decreased hypoxia-induced cardiac cell death, as detected by EthD-III staining (Figure 3(a)). Moreover, LDH levels were significantly increased in cardiac cells exposed to hypoxia and were decreased by melatonin administration (Figure 3(b)). As shown in Figure 3(c), the level of GSDMD-N was significantly increased after hypoxia exposure compared with the control; however, this increase was reversed by melatonin treatment. In addition, the levels of NLRP3 and cleaved caspase-1 were increased in cardiac cells after hypoxia exposure and were significantly decreased by melatonin treatment (Figures 3(d) and 3(e)). A similar change in NLRP3 was observed by immunofluorescence (Figure 3(f)). We also detected the effect of melatonin on IL-1*β* and IL-18 in H9C2 cells exposed to hypoxia. As shown in Figure 3(g), the protein levels of IL-1*β* and IL-18 were increased by hypoxia and reduced by melatonin administration.

### 3.4. Melatonin Attenuates NLRP3 Inflammasome-Mediated Pyroptosis by Inhibiting the TLR4/NF-*κ*B Signaling Pathway Both *In Vivo* and *In Vitro*

**Figure 4:**
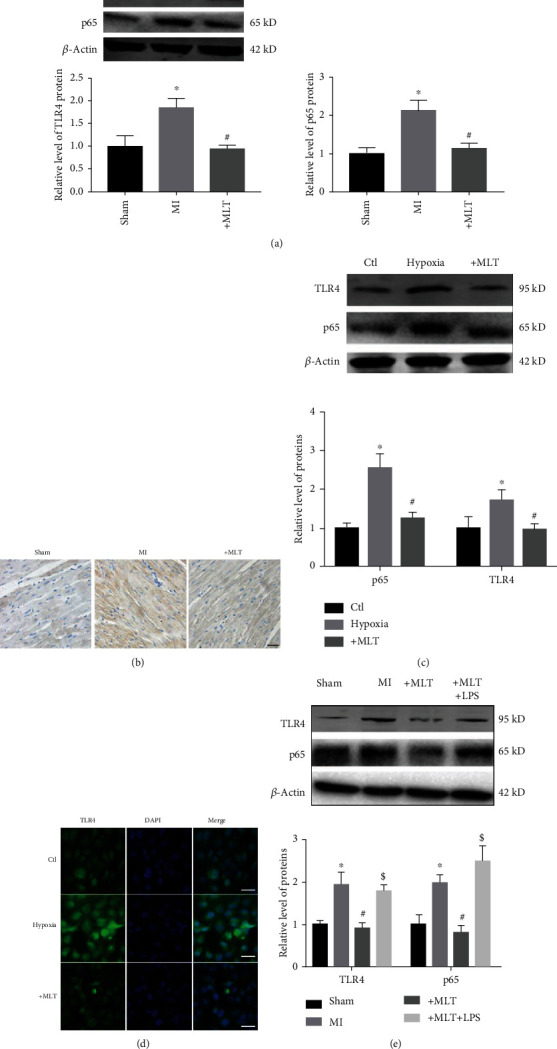
Effect of melatonin on the TLR4/NF-*κ*B signaling pathway both *in vivo* and *in vitro*. (a) Melatonin treatment reduced the levels of TLR4 and p65 in the hearts of MI mice. ^∗^*P* < 0.05 vs. sham, ^#^*P* < 0.05 vs. MI. Number of trials = 4. (b) Representative staining of TLR4 by immunohistochemistry. Scale bar, 20 *μ*m. (c) The levels of TLR4 and p65 were decreased in H9C2 cells by melatonin treatment. ^∗^*P* < 0.05 vs. ctl, ^#^*P* < 0.05 vs. hypoxia. Number of trials = 3. (d) TLR4 levels were detected by immunofluorescence. Scale bar, 50 *μ*m. (e) The protein levels of TLR4 and p65 in the hearts of MI mice with or without LPS treatment (2 mg/kg). ^∗^*P* < 0.05 vs. sham, ^#^*P* < 0.05 vs. MI, ^$^*P* < 0.05 vs. MLT. Number of trials = 4. MLT: melatonin.

Previous studies have demonstrated that the Toll-like receptor 4 (TLR4)/nuclear factor-*κ*B (NF-*κ*B) signaling pathway not only plays a critical role in MI-induced myocardial inflammation but also is involved in the NLRP3 inflammasome-mediated inflammatory response [32, 33]. Therefore, we investigated whether the antipyroptotic effect of melatonin in MI is associated with the TLR4/NF-*κ*B signaling pathway. As shown in Figures 4(a) and 4(b), the protein levels of TLR4 and NF-*κ*B (p-65) were increased in the hearts of mice following MI, and these increases were reversed by melatonin treatment. Consistent with the *in vivo* data, hypoxia also induced increases in TLR4 and p-65 levels in H9C2 cells, and melatonin administration significantly downregulated these hypoxia-induced increases (Figure 4(c)). A similar change in TLR4 was observed by immunofluorescence assay (Figure 4(d)). Lipopolysaccharide (LPS) is a common agonist of the TLR4/NF-*κ*B signaling pathway [34, 35]. Interestingly, we found that the effect of melatonin on the TLR4/NF-*κ*B signaling pathway was blocked by LPS (Figure 4(e)).

**Figure 5:**
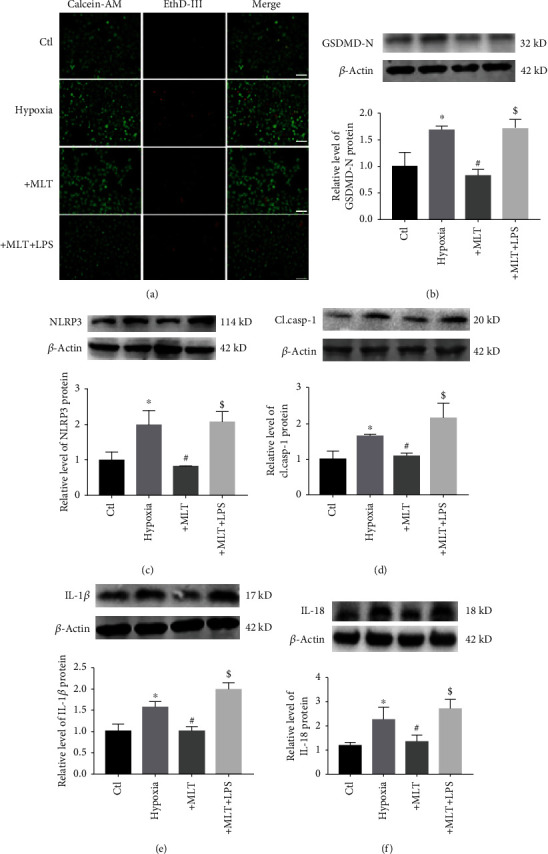
Effect of melatonin on NLRP3 inflammasome-mediated pyroptosis through inhibition of the TLR4/NF-*κ*B signaling pathway. (a) LPS (1 *μ*g/mL) blocked the effect of melatonin on H9C2 cardiomyocyte cell death. Live cells were stained with calcein-AM (green), and dead cells were stained with EthD-III (red). Scale bar, 40 *μ*m. (b–f) Representative images and statistical analysis of GSDMD-N (b), NLRP3 (c), and cl.casp-1 (d), mature IL-1*β* (e), and mature IL-18 (f) data from different groups. ^∗^*P* < 0.05 vs. ctl, ^#^*P* < 0.05 vs. hypoxia, ^$^*P* < 0.05 vs. MLT. Number of trials = 3. MLT: melatonin.

We also found that LPS treatment abrogated the ability of melatonin to reduce hypoxia-induced cardiomyocyte loss (Figure 5(a)). Importantly, as shown in Figures 5(b)–5(f), the antipyroptotic effect of melatonin was blocked by LPS treatment, as evidenced by decreased GSDMD-N, NLRP3, cleaved caspase-1, and mature IL-1*β* and IL-18 levels in H9C2 cells exposed to hypoxia. These data suggest that melatonin, at least in part, attenuates NLRP3 inflammasome-mediated pyroptosis by inhibiting the TLR4/NF-*κ*B signaling pathway.

## 4. Discussion

In the present study, we observed that melatonin ameliorated cardiac damage in an MI mouse model and hypoxia-induced cardiomyocyte injury in H9C2 cells. We also demonstrated that melatonin exerted cardioprotective effects by inhibiting cardiomyocyte death induced by NLRP3 inflammasome-mediated pyroptosis at the cellular level. The antipyroptotic effect of melatonin can likely be attributed to suppression of the TLR4/NF-*κ*B signaling pathway. These findings provide further support for melatonin as an effective agent for MI treatment.

MI-induced cardiac damage is a major cause of death worldwide and is mainly attributed to cardiomyocyte loss. Because cardiomyocytes have little potential for division, preventing cardiac cell death is considered an effective therapy for the treatment of MI. Numerous studies have found that myocardial inflammation is an important factor for cardiac dysfunction in MI progression [36, 37]. Inhibiting the inflammatory response is a potential strategy to reduce the loss of cardiomyocytes during MI [38–40]. Pyroptosis is inflammation-related cell death that has been shown to be involved in cardiomyocyte loss in cardiovascular disorders, such as MI and ischemia/reperfusion injury [41, 42]. Similar to previous studies, our present study found that the level of GSDMD-N, a pyroptosis executor, was significantly increased in the hearts of MI mice and in hypoxia-treated H9C2 cardiac cells. Moreover, the LDH assay data showed that MI induced marked pyroptosis-related morphological changes. However, strategies for preventing the loss of cardiomyocytes induced by pyroptosis in the progression of MI remain unclear.

Melatonin is a neuroendocrine hormone secreted by the pineal gland. Numerous studies have demonstrated that melatonin has beneficial effects in cardiovascular disorders, including MI, ischemia/reperfusion injury, atherosclerosis, heart failure, and hypertension [43, 44]. Similar to the results of previous studies, we found that melatonin administration significantly ameliorated cardiac dysfunction by normalizing the EF and FS values and reducing the number of inflammatory cells in the hearts of MI mice. Therefore, we next considered the mechanism by which melatonin prevents cardiac dysfunction. Previous studies have demonstrated that the underlying mechanisms of the cardioprotective effects of melatonin in MI are related to its abilities to inhibit the inflammatory response, decrease oxidative stress, maintain mitochondrial function, reduce cardiomyocyte apoptosis, and regulate receptor or nonreceptor signaling pathways (such as the MAPK/ERK, AMPK, PI3K/Akt, SAFE, and Notch pathways) [45–47]. However, whether the cardioprotective role of melatonin in MI is related to cardiac pyroptosis inhibition has not yet been proven. The results herein show that melatonin can significantly inhibit cardiac pyroptosis, as shown by the decreased GSDMD-N levels in both the hearts of MI mice and H9C2 cells exposed to hypoxia. Meanwhile, the levels of NLRP3, cleaved caspase-1, and mature IL-1*β* and IL-18 were markedly reduced by melatonin treatment both *in vivo* and *in vitro*. These data are the first to show that melatonin could prevent cardiac injury in part by inhibiting NLRP3 inflammasome-mediated cardiac pyroptosis.

TLR4, the first characterized mammalian Toll-like receptor, is widely accepted to play an important role in facilitating inflammatory responses during cardiovascular disease [48, 49]. For instance, TLR4 and its downstream gene NF-*κ*B were induced in the hearts of mice following MI [50]. Treatment with siRNA against TLR4 or a TLR4 inhibitor significantly reduced infarct size and restored cardiac function in MI mice [51]. The cardiomyocyte injury induced by hypoxia was inhibited by silencing TLR4 [52]. Moreover, the TLR4/NF-*κ*B signaling pathway has been shown to function upstream of the NLRP3 inflammasome during MI [53, 54]. However, whether the beneficial effect of melatonin on NLRP3 inflammasome-mediated pyroptosis is associated with the TLR4/NF-*κ*B signaling pathway in MI is still unclear. As predicted, we found that the activation of TLR4 and NF-*κ*B (p-65) in MI was inhibited by melatonin both *in vivo* and *in vitro*, whereas administration of LPS, an agonist of the TLR4/NF-*κ*B signaling pathway, abrogated the beneficial effect of melatonin on cardiac pyroptosis.

## 5. Conclusion

Taken together, our present study provides strong evidence that melatonin modulates MI-associated NLRP3 inflammasome-mediated cardiac pyroptosis by affecting the TLR4/NF-*κ*B signaling pathway, suggesting its utility in the treatment of MI.

## Data Availability

The data used to support the findings of this study are available from the corresponding author upon rational request.
